# Understanding the cognitive cost of multimedia learning: effects of visual load and language proficiency

**DOI:** 10.1186/s41235-025-00699-2

**Published:** 2026-01-06

**Authors:** Cintia Bali, Buket Tasdelen, Szabolcs Bandi, András Zsidó

**Affiliations:** 1 Department of Cognitive and Evolutionary Psychology, Faculty of Humanities and Social Sciences, Institute of Psychology, University of Pécs, Pécs, Hungary; 2 Contemporary Challenges Research Center, University of Pécs, Pécs, Hungary; 3 Department of Psychiatry and Psychotherapy, Clinical Centre, University of Pécs, Pécs, Hungary

**Keywords:** Cognitive theory of multimedia learning, Foreign language skills, Digital presentations, Sustained attention, Working memory capacity

## Abstract

Multimedia learning environments require learners to process and integrate information across visual and auditory modalities, often under conditions of limited cognitive capacity. In this study, we examined how visual load (defined as the number of images accompanying audio narration) and individual differences in language proficiency, sustained attention, and working memory influence learning outcomes in international university students. In two experiments (*N* = 61, *M* = 21.2 years), we examined how different visual loads affected memory recall. In Experiment 1, participants viewed narrated slides that included varying numbers of images, specifically from 0 to 3 images, and then completed an immediate recall task. In Experiment 2, we compared recall performance for audio-only vs. audio-and-picture information across two visual load conditions (1 vs. 3 images). Results showed that increasing visual support enhanced the learning of audio-and-picture information but had no benefit for audio-only content. Additionally, lower English proficiency and reduced attention were associated with poorer recall, especially under higher visual load. These findings support cognitive load theory and highlight how individual cognitive and language abilities can limit effective multimedia learning. The implications of these findings are discussed in relation to the design of digital instructional materials tailored for diverse learner populations.

## Significance statement

In real-world educational settings, poorly designed digital content that contains too many or irrelevant images can overwhelm learners and reduce their ability to understand and remember important information. This study explores these challenges by examining how visual load (the number of images), language proficiency, and sustained attention affect students’ recall of information from multimedia presentations. Our results indicate that including relevant images can enhance memory for combined audio-visual information, but it may not help the recall of simultaneously presented audio-only content (information presented solely through spoken words, without pictures). Additionally, students with lower language proficiency and attention performed poorly under higher visual loads, highlighting the risk of cognitive overload. These findings offer practical guidance for designing digital learning materials that balance visual support with learners’ cognitive and linguistic abilities, ultimately aiming to improve educational outcomes for diverse student populations.

## Introduction

### Advantages of learning with multimedia

In the contemporary era, higher education is significantly influenced by digitalization, presenting further challenges for educators and academics. This is evidenced by the increasing popularity and scientific interest in blended learning, e-learning, or multimedia learning approaches (Bizami et al., 2023). In the classroom, digital presentations (a series of slides that include text, images, video, and other multimedia elements to convey information) are often used as the preferred mode of delivery (James et al., 2006). Both teachers and students consider these digital presentations useful, informative, and captivating (Ravi & Waswani, 2020; Tang & Austin, 2009). Further, digital presentations have a great potential to enhance learning by utilizing the principles of multimedia learning. The *cognitive theory of multimedia learning* (CTML) (Mayer, 2002) posits that combining verbal and visual information enhances learning efficacy. Therefore, more efficient learning is expected from using digital presentations with multimedia elements (such as figures, videos, animations, and graphs). However, despite the expected benefits, there are mixed results regarding their effectiveness (Baker et al., 2018; Bali et al., 2025; Hoyt et al., 2024), meaning that the added value of these tools remains unclear. These mixed results pose a further challenge for educators and academics, who may already lack the requisite knowledge and confidence to utilize this method of delivery (Burke & James, 2008; Gordani & Khajavi, 2020; Seth et al., 2010; Sharp et al., 2017). This is especially true for students not learning in their native language whose need regarding the advantageous educational techniques is relatively unexplored (Macaro et al., 2018). Consequently, in this study, we aim to test the effectiveness of digital presentations with multimedia elements in order to provide specific suggestions on how to successfully promote learning, with a focus on international students. We believe that these suggestions can help teachers and academics to benefit more from the use of multimedia.

Digital presentations have the potential to facilitate learning by making it easy to incorporate multimedia elements into classroom learning. Multimedia elements can illustrate and complement the information provided in the classroom, leading to better learning, and understanding of abstract concepts (Kulasekara et al., 2011; Langer et al., 2021). Another advantage of multimedia learning is that it is an active form of learning that requires a higher level of cognitive engagement from students (Mayer, 2002). Consequently, it facilitates deeper comprehension and more efficient learning (Bujak et al., 2013; Jägerskog et al., 2019; Mayer & Moreno, 2002). This is particularly true for explanative multimedia elements, which are designed to demonstrate a process or illustrate how something works (Mayer et al., 1995). Additionally, multimedia elements play an important role in orienting attention and information selection (Bali & Zsido, 2024; Takacs & Bus, 2016). It can also be argued that multimedia elements may grab and hold students’ attention (Richter & Courage, 2017). These elements are often interesting and entertaining, which can lead to more focused attention and, thus, improved learning (Hidi, 1990; Renninger et al., 2014). This is especially crucial today because the immersed technological environment can lead to habituation to higher levels of environmental stimulation. As a result, more traditional face-to-face delivery modes may become less interesting and engaging to students (Nikkelen et al., 2014). The various ways of utilizing multimedia clearly show the multifaceted applications of these elements in the promotion of learning.

### Cognitive load in multimedia learning

Despite its well-documented effectiveness, there are certain limitations associated with multimedia learning. If not used thoughtfully, multimedia elements can be a source of unnecessary cognitive load (Sweller, 2012; Wiley et al., 2014). Such extraneous cognitive load often occurs when the multimedia elements are not related to the content or synchronized with the verbal information, which is called the congruency principle (Mayer & Moreno, 2003; Moreno & Mayer, 1999). Another fundamental principle of multimedia learning is that humans have a limited capacity to process information simultaneously. Therefore, presenting too many elements on the screen (regardless of whether they are related to the content) can cause cognitive overload and reduce the quality of information processing (Ayres & Sweller, 2014; Mayer, 2002). While some constraints are relatively easy to address (e.g., synchronizing or the use of content-related elements), less is known about when the amount of multimedia elements becomes overwhelming. Previous research has focused primarily on the disruptive effects of seductive (i.e., unrelated to the learning material) multimedia elements (Harp & Mayer, 1998; Sanchez & Wiley, 2006; Sundararajan & Adesope, 2020). However, recent studies have shown that even content-related elements can become distracting and interfere with learning when presented in large numbers (Makransky et al., 2021; Parong & Mayer, 2018; Plass & Kalyuga, 2019). Students learning through their second language may be even more affected as they have an inherently higher cognitive load (Roussel et al., 2017). Despite this, there is a lack of data on how to define excessive multimedia use, making it difficult to adapt to this constraint. Therefore, our goal is to provide clear recommendations on the effective use of images for multimedia learning.

### Individual differences in multimedia learning

Individual differences in cognitive processes are likely to contribute to the threshold at which cognitive overload from multimedia elements occurs. Foreign language proficiency is emerging as a new individual factor influencing the success of multimedia learning as the number of international courses and international students is increasing (Rienties et al., 2012; Singh et al., 2022). On the one hand, multimedia elements can certainly be useful for international students (Stiller & Schworm, 2019). On the other hand, they are at a higher risk of cognitive overload, as the use of a second language in itself requires more cognitive effort compared to the use of the native language, which is largely automatic (Roussel et al., 2017). This may reduce the effectiveness of multimedia learning and should be considered when designing multimedia learning materials. For second language learners, processing multiple multimedia elements simultaneously may be more demanding due to the already higher cognitive load, although it can be assumed that the level of foreign language proficiency may reduce this effect (Cloate, 2016). Recent studies have already emphasized that multimedia learning principles may differ for students learning in their second language (Kozan et al., 2015; Lee & Mayer, 2018); however, this area is relatively unexplored (Macaro et al., 2018). Given the higher risk of cognitive overload, the instructional design of a digital presentation with multimedia should be approached differently for international students. Therefore, in our study, we focused on international students in higher education and the instructional design that meets their needs.

In addition to foreign language proficiency, cognitive mechanisms such as working memory capacity and attentional mechanisms may also contribute to successful multimedia learning. Working memory (WM) capacity is the limited ability to temporarily store and manipulate the information required for complex cognitive processes such as reasoning, comprehension, and learning. Learners with a higher WM capacity can retain relevant information while processing new material, facilitating the formation of coherent mental representations, and supporting a deeper level of understanding (Conway et al., 2003). Sustained attention, in turn, is the ability to maintain focus on a task or stimulus over prolonged periods. It enables learners to process incoming information continuously and resist distraction, both of which are essential during extended instructional activities such as (Kokoç et al., 2020). Together, WM capacity and sustained attention provide complementary foundations for learning. WM governs the capacity to actively manipulate and integrate information, while sustained attention ensures the learner remains engaged for long enough to allow these cognitive processing to occur effectively. Consequently, differences in either domain can significantly impact learning outcomes, particularly in environments requiring continuous information processing and mental effort.

Learning with multimedia is a cognitively complex process; therefore, the role of individual differences might be even more pronounced—even when the instructional design of digital presentations follows the principles of multimedia learning (Mayer, 2002). Digital presentations require students to simultaneously process and integrate verbal and multiple visual information, but they have limited cognitive capacity to do so (Desimone & Duncan, 1995; Engle et al., 1999; Kane et al., 2007). Therefore, students with more limited WM capacity may have difficulties with processing all the information simultaneously, leading to early onset of cognitive overload and poorer comprehension of verbal and visual information (Sanchez & Wiley, 2006). In addition, when students exhibit higher distractibility and short attention spans, their information processing may become more fragmented, as some elements may capture their attention more than others (Colflesh et al., 2007). This can hinder simultaneous information processing and prevent meaningful learning by reducing the level of integration achieved between the verbal and visual information presented (Bali et al., 2025).

Recent research highlights that learners with higher working memory capacity or stronger attentional control are generally more resistant to distraction in multimedia environments (Bali et al., 2023a, 2023b; Lawson & Mayer, 2024a, 2024b, 2025; Makransky et al., 2021; Sanchez & Wiley, 2006; Wiley et al., 2014). Although such effects have often been demonstrated in studies using seductive, non-essential details, content-relevant multimedia elements may also increase cognitive demands when presented in greater numbers (Makransky et al., 2021; Parong & Mayer, 2018). Previous studies clearly indicate that better WM capacity reduces the disruptive effect of seductive details; however, little is known about the role of WM capacity and attentional mechanisms when only content-related elements are presented. The mixed results (Baker et al., 2018; James et al., 2006) call for further investigation as individual differences may partly explain them. Discovering the connection between effective multimedia learning and individual differences in core cognitive functions can help create digital presentations that fit better the needs of the audience.

### Aims of the study

The objective of this study is to test how incremental increases in visual elements affect information processing and recall within a specific multimedia learning environment. We assume that recommendations to achieve efficient information processing may vary based on individual differences in WM capacity and attentional processes. Consequently, the present study sought to examine the impact of varying amounts of explanative multimedia elements on the recall performance of university students while considering individual differences in attentional mechanisms, working memory capacity, and language proficiency. Understanding individual differences is essential for tailoring digital presentations to meet the needs of students. Based on these aims of our study, we developed the following hypotheses:

(1) An increasing number of visual items will lead to a gradual improvement in recall performance, although this improvement is expected to vary depending on individual differences in cognitive processes.

(2) Students with less efficient attentional processes and more limited WM capacity, recall performance will decline when more visual elements are presented on the screen.

(3) The same results will occur for foreign language proficiency as the cognitive load is inherently higher for those learning in their second language. Therefore, we hypothesize that students with lower levels of English proficiency will recall less information from the presented topic as the number of multimedia elements increases.

To test these hypotheses, we conducted two experiments. Experiment 1 investigated how the number of visual multimedia elements per slide and individual differences affects university students' learning outcomes. To gain a deeper understanding of the results, we conducted a complementary study (Experiment 2). The purpose of this experiment was to determine whether an increasing number of elements help participants achieve a more comprehensive understanding of the learning material or whether it primarily enhances knowledge acquisition through the visualization of information. In other words, whether participants simply remember information better when it is highlighted with pictures. Consequently, the use of more pictures may result in a larger portion of the learning material being emphasized through illustrations (for more see chapter: 3. Experiment 2).

## Experiment 1

Experiment 1 was designed to explore the impact of various quantities of explanatory multimedia elements on the recall performance of international university students, while also considering individual variations in attentional mechanisms, working memory capacity, and language proficiency. Participants’ recall performance was compared across four conditions, with the number of multimedia elements varying from 0 to 3.

### Methods

#### Sample

We recruited a total of 34 undergraduate psychology students (23 women, 3 preferred not to answer) studying in the English program between the ages of 19 and 37 (*M* = 22, *SD* = 3.90). Participants studied in an English program; therefore, during the application process, they were screened for language proficiency and had at least a B2-level English language certificate. All the participants were healthy adults, and none of them reported having a psychiatric disorder. Participation was voluntary and they did not receive compensation for their participation. Data collection was carried out during university seminars. The study was approved by the Hungarian United Ethical Review Committee for Research in Psychology (reference nr. 2023-104) and was carried out following the Declaration of Helsinki. We obtained informed written and verbal consent from all participants. For the detailed descriptive data see Table 1.

**Table 1:** Mean scores and standard deviations (SD) of the retention test, the attentional skill scores (*E*%), the English proficiency scores, and the digit-span task scores (WM capacity). Retention test scores are presented in total and by conditions

Task		Mean	SD
Retention test	Control	3.59	1.84
	Multimedia1	3.37	1.79
	Multimedia2	4	1.72
	Multimedia3	4.44	1.91
	Total	3.85	1.85
*Cognitive tasks*			
Attentional skill (D2)	E%	7.5	4.98
English proficiency	Language	3.63	1.7
Digit-span task (backward)	WM capacity	6.76	1.26

#### Instruments

##### Presentations

During data collection, participants viewed a short multimedia presentation designed to introduce Cloninger’s psychobiological theory (Cloninger, 1987; Cloninger et al., 1998; Serretti et al., 2006). The topic was selected in consultation with seminar instructors to ensure that it aligned with the syllabus but was unfamiliar to the students. As a result, we created a presentation featuring Cloninger’s psychobiological theory. The presentation consisted of 16 PowerPoint slides, each accompanied by narration. Slides contained 0, 1, 2, or 3 visual multimedia elements (e.g., figures, static images, or GIFs); the number of elements defined the four experimental conditions used in the study. The elements were relevant to the presented content and followed established multimedia learning principles (Lee & Mayer, 2018; Mayer & Moreno, 2003; Moreno & Mayer, 1999) to eliminate any potential confounding effects on cognitive load unrelated to the number of displayed elements.

**Fig. 1:**
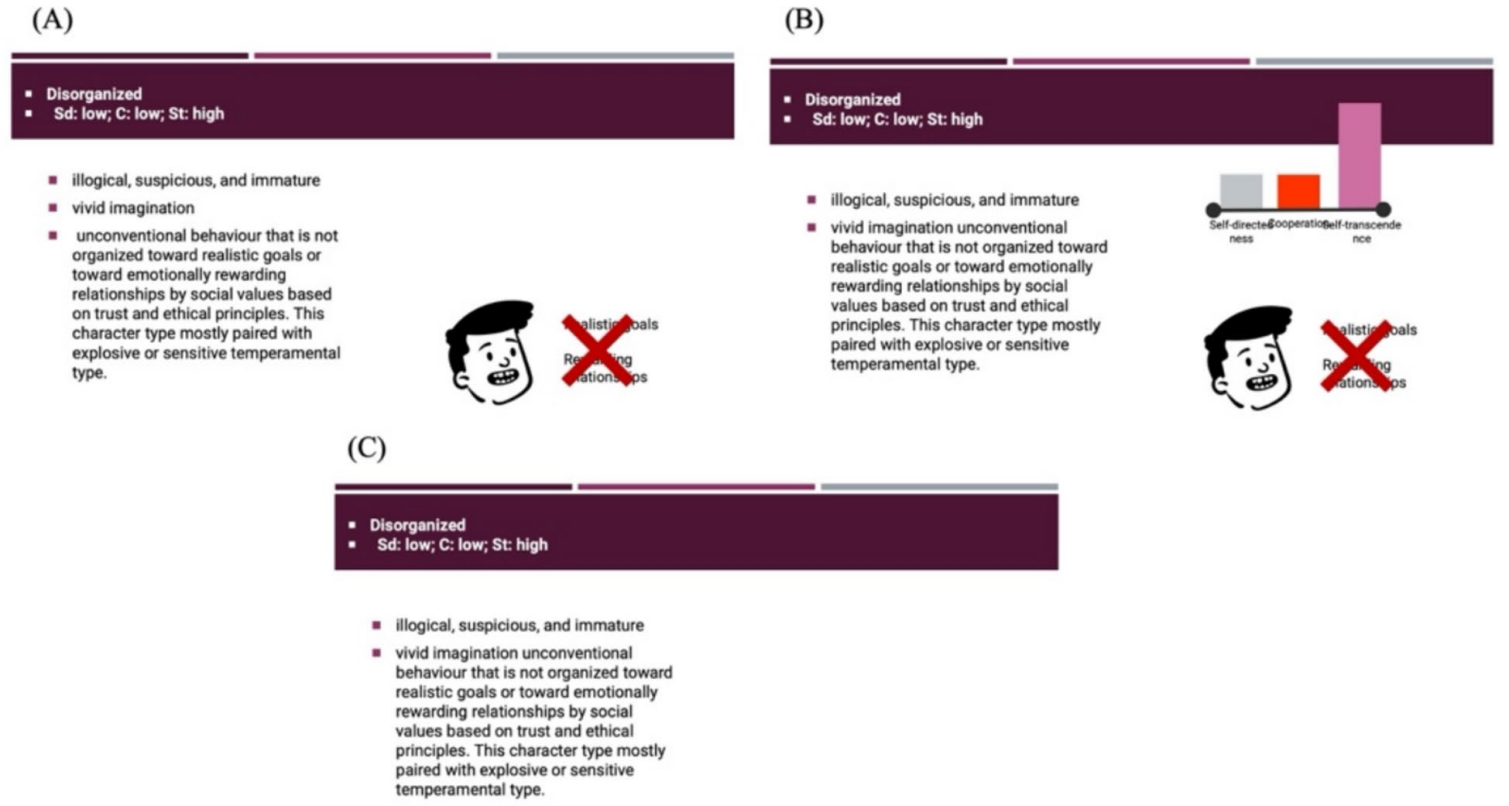
An example slides from the presentations used in the study. The same slide from each of the versions. **A** represents the condition with one multimedia element, **B** shows the condition with two multimedia elements, and **C** represents the control condition with no multimedia elements

Each participant was exposed to all four multimedia conditions (0, 1, 2, or 3 visuals per slide), making this a within-subjects design. The presentation included four slides for each condition (4 × 4 = 16 slides). To prevent potential content effects from specific slides, we created three counterbalanced versions of the presentation. In these versions, the same slides and narration were used; the only difference among the three versions of the presentation was the number of multimedia elements assigned to specific slides. For instance, a slide featuring one visual element in Version A had two elements in Version B and no elements in Version C. Figure 1 illustrates how a slide changed across the three versions based on the experimental conditions. This counterbalancing ensured that any observed differences in learning performance could not be attributed to the specific slide content or the difficulty of the slide topics, but rather to the experimental manipulation—the number of visual multimedia elements presented.

The presentations were independently evaluated by three research assistants and the three seminar instructors before data collection began. They assessed the relevance and adequacy of the visuals in supporting the lecture content. Based on their feedback, several adjustments were made to ensure that the visuals were well aligned with both the spoken and written materials.

##### Retention test

To measure the learning outcome, we asked participants to answer multiple-choice questions related to the presented topic (e.g., *‘Which of the following is not true for novelty seeking?’*). We handed out the retention test immediately after the presentation. During the evaluation of recall performance, we organized the retention test questions into four sets of eight items, each set corresponding to one multimedia condition (0, 1, 2, or 3 visuals). This allowed us to assign a retention test score for each condition to every participant. All participants answered the same 32 questions; however, the calculation of the retention test scores was adjusted according to the experimental condition to which each question referred. Thus, depending on the presentation version a participant viewed, the same question could assess recall performance for, for example, the one-picture condition or for the three-picture condition. Participants received one point for each correct answer and 0 for an incorrect answer; they could achieve a maximum of 32 points (i.e., 8 points per condition) by answering all of them correctly.

##### Attentional skill

We asked the participants to complete the d2 Test of Attention (Brickenkamp & Zillmer, 1998) to measure their sustained and selective attentional skills. The d2 is a paper-and-pencil cancelation task that requires high concentration and resistance to fatigue. It consists of a test sheet that portrays overall 658 “p” and “d” letters across 14 lines, each with 47 letters. The letters are surrounded by one to four dashes arranged below or above the figures. Participants had to find and cancel as many targets as they could within 20 s per line. The time was measured by the experimenter. After hearing the stop signal, participants had to stop and draw a straight line at the last attended figure of the given line and then move on to the next line immediately. Overall, the task lasted about five minutes. To evaluate the performance of the participants, the total number of attended figures (N) and the total number of errors (E) (canceled non-target figures and omissions) were counted. We used these values to calculate the percent of errors (E%) using the following equation ((E/N*100)). Higher scores indicate worse performance.

##### Working memory capacity

We used the backward version of the digit-span task (Jones & Macken, 2015) to measure working memory capacity. Participants were shown 15 sequences of digits one after another on the screen in the classroom. They had to observe each sequence carefully and then write them down on a blank paper in reverse order. The number of digits increased by one after every two sequences. Participants saw the first pair of sequences for two seconds; the presentation time was then increased by half a second per digit. Before the task participants were shown one sequence as a trial. The answers were evaluated until the participant had made at least two consecutive errors. The length of the last correctly recalled sequence was used as an indicator of working memory capacity. Higher scores indicate greater WM capacity. Participants could achieve a total of nine points.

##### English proficiency

Since English was not the native language of our participants, we screened for their English proficiency using a C1-level comprehension test from a TELC (The European Language Certificates) mock language examination. We asked the participants to read a short text and fill in the missing sentences. They received one point for each correct answer; thus, they could achieve a maximum of six points.

#### Procedure

The experiment took place during personality psychology seminars for undergraduate psychology students in the English BA program after a prior agreement with the teachers and students. We visited three seminar groups (12, 8, and 14 participants, respectively), each viewing a different version of the presentation. Participants in the same seminar attended simultaneously and watched the presentation on a television screen in the classroom, viewing the slides in the same order. To ensure a balanced representation of the multimedia conditions, we utilized three different versions of the presentation across the three seminar groups. Thus, while all participants received the same information, the sequence of conditions (0, 1, 2, or 3 visual multimedia elements) varied between seminar sessions.

First, the students who attended the seminar received an informed consent form. The experimenter emphasized that participation is voluntary and there are no negative consequences of withdrawal from the study. Participation required the written consent of the students. If the students agreed to participate, we handed out the test battery and asked the students to complete the first page consisting of the demographic questions. Afterward, the first author presented the slides on the 55-inch televisions placed in the classrooms. Immediately after the presentation, we asked the students to fill in the retention test according to their best knowledge. When participants finished the retention test, they completed the backward digit-span task, the d2 test of attention, and the English proficiency test. The whole experiment lasted about 1-h.

#### Data analysis

Statistical analyses were performed using the ‘lme4’ (Bates et al., 2015) and ‘emmeans’ packages in R (version 2023.09.1 + 494). All variables were normally distributed, as the absolute values of Skewness and Kurtosis were less than 2. Participants with missing values in the cognitive variables were excluded (approximately 17% of all the collected data). E% scores, achieved points on the TELC comprehension test, and digit-span scores were transformed into z-scores and centered at zero.

We sought to test the effect of the number of multimedia elements on the student’s performance on the retention test. For this, we performed a random-intercept linear mixed model (lmm), where the within-subject factor was the number of multimedia elements (0 to 3). Achieved scores on the retention test were included as dependent variables. Individual differences in WM capacity, attentional performance, and English language proficiency were included as independent predictors. We tested the main effects and interactions between the within-subject factor (number of multimedia elements) and the backward digit-span scores (WM capacity), E% scores (sustained attention), and the achieved points on the TELC comprehension test (language). The random factor was the participants' code. Statistical results will be presented in a table to make the description of the results easier to follow.

The dataset that includes computed study variables is available on the Open Science Framework: https://osf.io/a7vh8/?view_only=736f6bcaa72d408fb3ace7ccee1d4aee

### Results and discussion

**Fig. 2:**
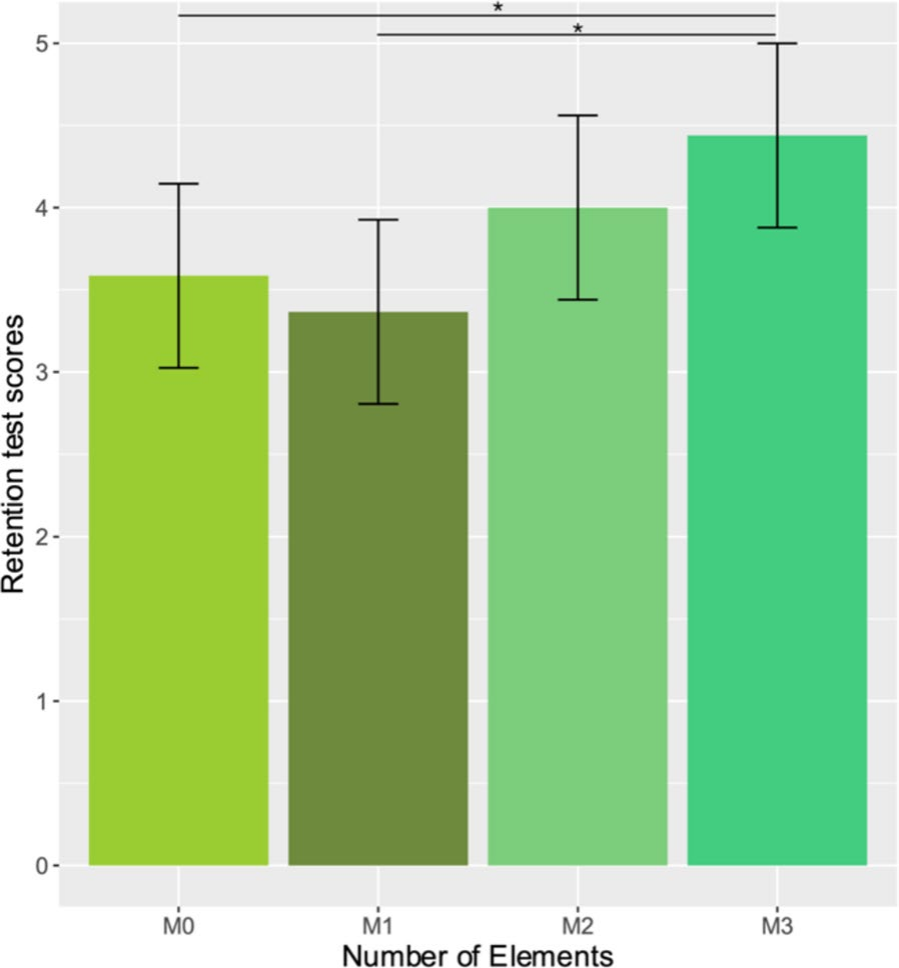
The students’ learning outcomes, represented by the mean scores on the retention test separated by the number of presented visual elements (M0 = no presented elements, M1 = one element, M2 = two elements, M3 = three elements). The error bars indicate the 95% confidence interval

The objective of Experiment 1 was to test whether the number of multimedia elements presented in a digital presentation would improve the learning outcomes of university students. Statistical results are presented in Table 2; see Fig. 2 for mean scores. We hypothesized that as the number of multimedia elements increased, students would remember the presented learning material better. In line with our hypothesis, the analysis revealed a significant main effect regarding the number of multimedia elements. Participants’ learning outcomes improved significantly when they were exposed to three visual multimedia elements compared to when they were exposed to none or one element during learning. This suggests that the increased number of multimedia elements does indeed gradually improve recall performance (see Fig. 2).

**Table 2:** Detailed statistical results for the linear mixed models with pairwise comparisons regarding the number of multimedia elements and the interactions between conditions and attention (*E*%), English proficiency (language), and WM capacity (backward digit-span scores). Significant interactions are broken down by condition. Significant main effects and interactions are italicized

		Fixed effects				
	b	95% CI		*df*	*t*	p
		Lower	Upper			
M1–M0	–0.176	–0.900	0.547	90	–0.478	0.634
M2–M0	0.471	–0.253	1.194	90	1.275	0.206
*M3–M0*	*0.765*	*0.041*	*1.488*	*90*	*2.071*	*0.041*
M2–M1	–0.647	–1.241	–0.053	90	–1.753	0.083
*M3–M1*	*–0.941*	*–1.791*	*–0.091*	*90*	*–2.549*	*0.012*
M2–M3	–0.294	–1.129	0.541	90	–0.797	0.428
*E*%	–0.271	–0.567	0.146	30	–1.160	0.255
Language proficiency	0.107	–0.271	0.494	30	0.571	0.572
WM capacity	–0.245	–0.607	0.109	30	–1.363	0.183
M0–M1*E%	*–1.134*	*–1.848*	*–0.367*	*90*	*–2.931*	*0.004*
M0–M2*E%	–0.489	–1.218	0.263	90	–1.263	0.210
M0–M3*E%	–0.547	–1.274	0.206	90	–1.414	0.161
M0–M1*Language proficiency	0.133	–0.656	0.932	90	0.341	0.734
M0–M2*Language proficiency	0.572	–0.199	1.390	90	1.469	0.145
M0–M3*Language proficiency	*1.335*	*0.596*	*2.184*	*90*	*3.430*	< 0*.001*
M0–M1*WM capacity	*0.870*	*0.140*	*1.627*	*90*	*2.33*	*0.022*
M0–M2*WM capacity	0.373	–0.365	1.122	90	0.999	0.321
M0–M3*WM capacity	0.681	–0.052	1.435	90	1.823	0.072

Random effect		
	Variance	SD
Subject (intercept)	0.495	0.703
Residual	2.317	1.522

Model fit		
	Marginal	Conditional
*R*^2^	0.222	0.359

model <—mixed(score ~ zD2 + zlanguage + zDigit_span + Elements + Elements*zD2 + Elements*zlanguage + Elements*zDigit_span + (1 | subject),data = STUDY1,control = lmerControl(optimizer = "bobyqa"), REML = TRUE)

Key: *p* values for fixed effects calculated using Satterthwaites approximations

**Fig. 3:**
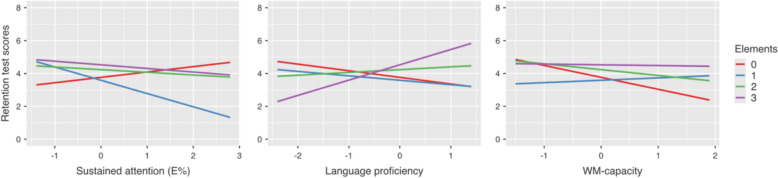
The relationship between participants' learning outcomes and their sustained attention scores (**A**), language proficiency (**B**), and WM capacity (**C**) separated by conditions (elements). The y-axis represents the retention test scores, with higher values indicating better performance. The x-axis shows the achieved scores in z-scores regarding the continuous predictor variables, where higher values correspond to greater inattention, better language proficiency, and greater WM capacity. Multiple lines are plotted to depict how this relationship varies among different conditions

Regarding individual differences, we tested the effect of sustained attention, WM capacity, and English proficiency on learning efficiency. We hypothesized that students with less efficient attentional processes and more limited working memory capacity would show reduced learning efficiency when more visual elements were presented on the screen. In the analysis, we also controlled for the English proficiency of the students, as the learning material was not delivered in their native language. We did not find a significant main effect of these variables; however, the analyses revealed significant interactions (see Fig. 3). The findings indicate that students with shorter attention spans tend to process information less effectively and recall less information accurately when multimedia elements are present. While a positive trend was observed in the control condition, we noted negative tendencies in the multimedia conditions, with a significant association in M1 (*t* (109.80) = − 2.68, *p* = 0.008). We found similar negative tendencies for language proficiency; however, the association was only significant when students were exposed to three multimedia elements (*t* (109.80) = 3.07, *p* = 0.003). In terms of WM capacity regardless of the significant interaction, we found that students perform equally well despite the number of the presented multimedia elements. WM capacity and recall performance only showed significant associations in the control condition, where the design did not include multimedia elements (*t* (109.80) = − 2.49, *p* = 0.014).

Our findings offer valuable insights into the role of visual elements in multimedia learning. Nevertheless, the results raise several new questions and suggest alternative explanations that require further investigation. In the retention test, we used a mixture of questions about visually displayed (audio-and-picture information) and non-displayed information (audio-only information) presented during the digital presentations. As the number of visual elements increased, participants were exposed to a greater proportion of content that was reinforced both verbally and visually. The picture superiority effect suggests that individuals remember pictures better than words (Paivio & Csapo, 1973; Stenberg, 2006; Winograd et al., 1982) because pictures have a perceptual advantage due to their distinctive features (Mintzer & Snodgrass, 1999). Therefore, participants may have primarily processed and encoded visually presented information, leading to enhanced recall of audio-and-picture information.

If this interpretation is right, multimedia effect could be explained by the perceptual advantage of pictorial information. However, it is unclear from our results whether multimedia elements support this fragmented learning of the displayed information or facilitate a comprehensive understanding of the topic, as proposed by the CTLM (Mayer, 2014). Furthermore, we cannot conclude that multimedia elements decreased the overall encoding of information for students with weaker English skills, limited working memory capacity, and less effective sustained attention. It is also possible that these students may have lacked the cognitive resources necessary to process audio-only information because their attention was focused on detecting and integrating visual information. Therefore, experiment 2 was designed to address these questions.

## Experiment 2

A slide in a digital presentation typically visually displays some of the information but not all the content of the accompanying spoken information presented (James et al., 2006). This raises the question of whether students remember audio-only information as well as audio-and-picture information presented with the same slide. Information is considered audio-only when it is presented solely through spoken words, without any visual representation. In contrast, information is classified as audio-and-picture when it is accompanied by visuals that directly correspond to the spoken content, illustrating or reinforcing the information being conveyed audibly. The CTLM suggests that the combination of text and images in an educational context can facilitate meaningful learning through increased cognitive engagement, which is a consequence of active learning (Mayer, 2002). On this basis, we would expect multimedia elements to support global comprehension. However, it is also possible that the visual elements highlight certain content from the subject material and primarily support a fragmented learning rather than global comprehension (Stenberg, 2006). If multimedia elements support global comprehension, we would expect students to show better recall performance for both audio-and-picture and audio-only information. Conversely, if multimedia elements work by highlighting specific information and capturing attention through their distinctive features, only the learning of pictorial information would be enhanced. Regarding individual differences, visualization may also play an important role. Students with attentional difficulties might show impaired learning performance only for audio-only information during the short lecture. Since students with impaired attentional mechanisms face greater challenges with multisensory integration (Panagiotidi et al., 2017; Talsma et al., 2010), the picture superiority effect may be even more pronounced for them during simultaneous processing.

Compared to Experiment 1, the retention test in Experiment 2 included an equal number of questions about audio-and-picture and audio-only information. With this modification, in addition to the number of elements, we added the visualization of the information as a second within-subject factor. Compared to Experiment 1, we reduced the number of tested conditions regarding the number of multimedia elements and tested only one and three multimedia elements. This was motivated by the fact that it allows us to test learning effectiveness in a lower and higher load situation. Furthermore, the results of Experiment 1 suggest that three elements can induce significant improvements in learning compared to one element.

### Method

#### Sample

The sample consisted of 27 undergraduate psychology students (20 women) studying in the English program between the ages of 19 and 23 (*M* = 20.4, *SD* = 1.45). Sampling was identical to Experiment 1. For the detailed descriptive statistics see Table 3. All the participants were healthy adults, and none of them reported having a psychiatric disorder. Participation was voluntary and the students did not receive compensation for their participation. The study was approved by the Hungarian United Ethical Review Committee for Research in Psychology (reference nr. 2023-104) and was carried out following the Declaration of Helsinki. We obtained informed written and verbal consent from all participants.

**Table 3:** Mean scores and standard deviations (SD) of the retention test, the attentional skill scores (*E*%), the English proficiency scores, and the digit-span task scores (WM capacity). Retention test scores are presented in total and by conditions based on the number of elements and visualization

Task			Mean	SD
Retention task	n of elements	Multimedia1	4.43	1.62
		Multimedia3	4.1	1.75
	Visualization	Audio-and-picture	4.59	1.79
		Audio-only	3.95	1.54
	Total		4.27	1.69
Cognitive tasks				
Attentional skill (D2)		E%	7.72	5.05
English proficiency		Language	3.85	1.62
Digit-span task (backward)		WM capacity	6.74	1.22

#### Instruments

##### Presentations

In Experiment 2 we used slightly modified versions of the same presentation that we used in Experiment 1. The slides featured the same topic and were accompanied by the same narration, text, and multimedia elements. The presentation differed only in the number of multimedia elements. In Experiment 2 the number of multimedia elements that could appear on a slide was either 1 or 3 pieces, resulting in 2 conditions regarding the number of multimedia elements. We had eight slides with one and another eight slides with three multimedia elements. The number of multimedia elements varied randomly across the slides. To prevent potential content effects from specific slides, we created two counterbalanced versions of the presentation. In these versions, the same slides and narration were used; the only difference among the two versions of the presentation was the number of multimedia elements assigned to specific slides.

##### Retention test

To measure recall performance, we asked participants to answer multiple-choice questions related to the presented topic. We handed out the retention test immediately after the presentation. The test contained two questions referring to each slide, which resulted in a total of 32 questions. The questions can be divided into 4 (2 × 2) conditions (eight questions per each) along two dimensions. One dimension is the number of multimedia elements (1 or 3), and the other is whether the question asks for information visualized with a multimedia element or not. Information is visualized when illustrated with pictures on slides (referred to as audio-and-picture information) and is non-visualized when conveyed solely through written text and audio narration without accompanying pictures (referred to as audio-only information). Illustrations featured on the slides were always accompanied with audio information.

All participants answered the same 32 questions; however, the calculation of the retention test scores was adjusted according to the experimental condition to which each question referred. Thus, depending on the presentation version a participant viewed, the same question could assess recall performance for, for example, an audio-and-picture information with one picture or for an audio-and-picture information with three pictures. Participants received one point for each correct answer and 0 for an incorrect answer; they could achieve a maximum of 32 points (eight points per condition) by answering all of them correctly.

#### Procedure

The procedure was identical to Experiment 1.

#### Data analysis

Statistical analyses were performed using the ‘lme4’ (Bates et al., 2015) and ‘emmeans’ packages in R (version 2023.09.1 + 494). The participants who failed to complete the digit-span task were excluded (approximately 7% of all the collected data). All variables were normally distributed, the absolute value of Skewness and Kurtosis was less than 2. E% scores, achieved points on the TELC comprehension test, and digit-span scores were transformed into z-scores and centered at zero.

We sought to test the effect of the number of multimedia elements and visualization on the student’s performance on the retention test. For this, we performed a random-intercept lmm, where the within-subject factors were the number of multimedia elements (1 or 3) and the visualization of the conveyed information (audio-and-picture or audio-only). Achieved scores on the retention test were included as dependent variables. We included WM capacity, attentional performance, and English language proficiency as independent predictors to test whether these factors influenced the retention test scores. We tested the main effects and interactions between the within-subject factors (number of multimedia elements and visualization) and the backward digit-span scores (WM capacity), E% scores (sustained attention), and the achieved points on the TELC comprehension test (language). The random factor was the participants' code. Statistical results will be presented in a table to make the description of the results easier to follow.

The dataset that includes computed study variables is available on the Open Science Framework: https://osf.io/a7vh8/?view_only=736f6bcaa72d408fb3ace7ccee1d4aee

### Results and discussion

**Fig. 4:**
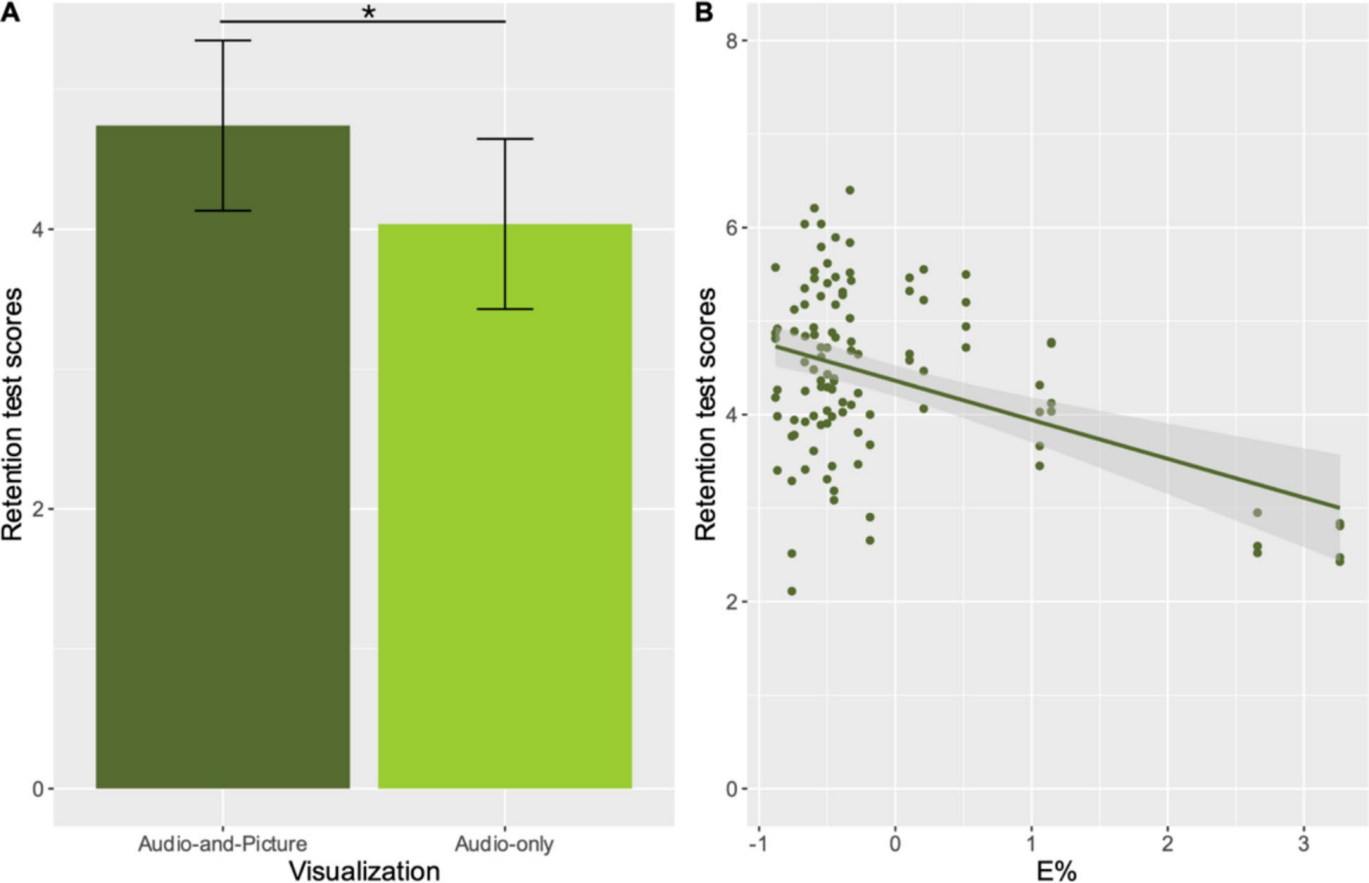
**A** Performance on the retention task (retention test scores = number of total points obtained) for the audio-and-picture information and audio-only information. **B** The association between the overall learning outcomes (retention test scores) and the sustained attention of the participants (*E*%). The y-axis represents the retention test scores, with higher values indicating better performance. The x-axis shows sustained attention scores transformed into z-scores, where higher values correspond to greater levels of inattentiveness

The aim of Experiment 2 was to test how the visualization of information affects the processing of audio-only information when students learn from a digital presentation with visual multimedia. The analyses showed no main effect of the number of elements; however, we found a significant effect of visualization (see Fig. 4). This confirms the assumption that visual multimedia elements in digital presentations primarily support the acquisition of audio-and-picture information during a short lecture. These results also highlight that for memory encoding visual representation can be more important than the number of elements presented on the screen. We did not find any interaction between the number of elements and visualization. This suggests that up to three visual multimedia elements do not interfere with the processing of audio-only information or at least not more than a single presented element. This is supported by the fact that students correctly recalled approximately the same amount of information from audio-only information whether one or three multimedia elements were presented. Statistical results are reported in Table 4.

**Table 4:** Detailed statistical results for the linear mixed models with main effect and interaction between within-subject factors. Interactions between conditions and attention (*E*%), English proficiency (language), and WM capacity (backward digit-span scores) are also reported. Significant main effects and interactions are italicized

		Fixed effects				
	*b*	*Df*	95%CI		*t*	*p*
			Lower	Upper		
M3–M1	−0.407	72	−0.908	0.094	−1.593	0.115
*Visualization (Audio-only–Audio-and-Picture)*	*−0.703*	*72*	*0.203*	*1.205*	*−2.753*	*0.007*
Elements*Visualization	0.074	72	−1.076	0.928	0.144	0.885
*E%*	*−0.059*	*23*	*−0.114*	*−0.006*	*−2.164*	*0.041*
Language proficiency	0.001	23	−0.266	0.268	0.007	0.994
WM capacity	−0.230	23	0.633	0.172	−1.12	0.274
M3–M1*E%	0.013	72	−0.053	0.079	0.395	0.694
M3–M1*Language	−0.229	72	−0.554	0.095	−1.38	0.170
M3–M1*WM capacity	−0.223	72	−0.723	0.257	−0.933	0.354
Audio-only–Audio-and-picture*E%	0.042	72	−0.108	0.024	1.255	0.213
Audio-only–Audio-and-picture*Language	0.189	72	−0.515	0.135	1.14	0.256
Audio-only–Audio-and-picture*WM capacity	0.104	72	−0.594	0.385	0.417	0.678

	Random effect		
		Variance	SD
Subject (intercept)		0.751	0.867
Residual		1.764	1.328

	Model fit		
		Marginal	Conditional
*R*^2^		0.165	0.414

model <—mixed(score ~ Elements * Display + Elements * zD2 + Display * zD2 + Elements * zlanguage + Display * zlanguage + Elements * zDigit_span + Display * zDigit_span + (1 | ID),data = ML2,control = lmerControl(optimizer = "bobyqa"), REML = TRUE)

Key: *p* values for fixed effects calculated using Satterthwaites approximations

Regarding individual differences the analysis revealed a main effect of sustained attention; however, we found no other significant main effect or interactions. The exact statistical results are shown in Table 4. Since no significant interaction with visualization was found for sustained attention and English proficiency, it can be assumed that there is a global decrease in learning efficacy associated with poorer sustained attention when multimedia elements are presented. This is evidenced by the fact that students with poorer sustained attention generally performed worse on the retention test. Thus, the observed effect of these variables in Experiment 1 (a decrease in Q&A scores when multimedia elements are included) is not limited to audio-only information. This finding is consistent with the interaction pattern observed in Experiment 1, where lower levels of sustained attention were associated with poorer performance in multimedia conditions. These results suggest that sustained attention is crucial for multimedia learning, as it helps regulate how learners allocate cognitive resources across different modalities. When attentional resources are insufficient, learners may struggle to effectively integrate visual and auditory information, resulting in reduced comprehension and recall.

## Discussion

### Empirical implications

Our study aimed to investigate the impact of visual multimedia elements used in digital presentations on information processing and learning. Specifically, we sought to test whether increasing the number of multimedia elements up to three would improve the learning outcomes of university students. Our results suggest that content-related explanative multimedia elements facilitate learning as the observed learning outcomes were highest with three elements. However, when including the visualization (audio-only or audio-and-picture) of the recalled information the number of multimedia elements seems to be less pronounced. Our results suggest that students primarily remember content that is presented both visually and verbally, and this improvement in learning is independent of the number of visually presented elements. This indicates that in Experiment 1 participants might recalled more information in the three-picture condition simply because there is more content visually represented compared to the one-picture condition. The results are in line with previous studies (Gordani & Khajavi, 2020; Lee & Mayer, 2018; Mayer, 2002) and confirm that the inclusion of explanative multimedia elements (in this case static illustrations) facilitates learning. However, the improvement in recall performance is not due to global comprehension and processing. Instead, more fragmented learning predominates, which might be explained by the fact that the visualization can highlight some specific content and capture attention (Mitzner et al., 2019).

In addition to the number of multimedia elements, we further examined the impact of individual differences in sustained attention, working memory (WM) capacity, and language proficiency across Experiments 1 and 2. Students with lower levels of sustained attention demonstrated poorer recall performance for both audio-and-picture and audio-only content. This suggests that when attentional processes are less efficient, multimedia elements can hinder overall understanding. This not only emphasizes the significance of examining individual differences (Li et al., 2019) but also demonstrates that explanative content-related multimedia elements can negatively impact learning outcomes when attentional processes are less efficient. In contrast, WM capacity did not significantly correlate with learning outcomes in multimedia conditions, indicating that—in line with the finding of Lawson and Mayer (2024a, 2024b)—sustained attention may be more crucial in regulating cognitive resources during multimedia learning. In Experiment 1, students with lower language proficiency performed worse when multiple pictures were shown. However, this effect was not observed in Experiment 2, which may be due to the greater homogeneity of the sample in the latter study, where participants generally exhibited higher levels of language proficiency.

### Theoretical implications

The current findings have several theoretical implications for the CTLM and related cognitive processing frameworks. While our results support the idea that explanatory visuals can improve recall, they also suggest that this effect may arise not from enhanced understanding but rather from the perceptual advantages of visual information. The pattern of results aligns with the picture superiority effect (Paivio & Csapo, 1973; Stenberg, 2006; Winograd et al., 1982). This effect suggests that images are remembered more effectively than words because of their unique perceptual characteristics (Mintzer & Snodgrass, 1999).

Students with shorter attention spans showed globally impaired learning efficiency, indicating that parallel processing increases cognitive demands, even with a relatively small number of elements. Previous studies have found this disruptive effect of multimedia elements primarily in the context of seductive (i.e., entertaining but unrelated to content) details (Harp & Mayer, 1998; Sanchez & Wiley, 2006; Wiley et al., 2014). A similar effect was observed for foreign language proficiency, indicating that it may be an important factor in determining the most appropriate instructional design for non-native language learners. As previously demonstrated (Lee & Mayer, 2018), different multimedia principles may apply to students who are learning in their second language. Our findings also suggest that, therefore, these students may warrant further interest in future research. It appears that learning with multimedia is more demanding with lower language proficiency, which affects students’ learning effectiveness in a multimedia environment. In our study a decrease in learning success was observed despite the fact that the presentations included written text in addition to the spoken information. Based on the modality effect (Ginns, 2005; Knoop-Van Campen et al., 2018; Moreno & Mayer, 1999; Tabbers et al., 2004) providing written text and spoken information simultaneously would increase cognitive load, but the opposite was observed for students learning in their second language (Kozan et al., 2015; Lee & Mayer, 2018). WM capacity showed no association with learning outcomes when multimedia elements were included. Students performed equally well despite the number of illustrations. This is somewhat surprising, as previous research in multimedia learning has mainly emphasized the role of WM capacity in the context of individual differences, while the possible contribution of sustained attention was not investigated (Anmarkrud et al., 2019; Doolittle & Mariano, 2008; Kozan et al., 2015; Sanchez & Wiley, 2006; Wiley et al., 2014). Thus, in the future, it may be worthwhile to include attentional processes in the study of multimedia learning, as it appears that attentional processes may contribute more to the prevention of cognitive overload.

### Practical implications

Beyond theoretical contributions, the results also provide practical implications for instructional design in higher education. Our findings indicate that explanatory multimedia elements can effectively emphasize key content in digital lectures; however, they should be used carefully. While most students can effectively process up to three visual elements, individuals with lower sustained attention may struggle to integrate visual and auditory information. For educators, this means that even content-related visuals can impede learning when attentional capacity is limited. Therefore, the instructional design of multimedia materials should take individual differences in attention and language proficiency into account. Adjusting the number and complexity of visuals to match learners’ cognitive profiles may help optimize learning efficiency and prevent increased cognitive load. These results can guide educators in creating multimedia presentations that adhere to multimedia learning principles while avoiding excessive visual stimulation that could overwhelm students' attentional resources.

### Limitations and future directions

Some limitations of the study should also be noted. Prior knowledge of Cloninger’s psychobiological theory was not formally assessed. However, we consulted with the seminar instructors, who confirmed that the students did not possess extensive prior knowledge on the topic. Importantly, since our study employed a within-subjects design, we compared each participant's performance across different conditions relative to their own baseline. Thus, while prior knowledge is always a factor to consider, it is less likely to have influenced the observed effects in this study. Also, we measured recall performance immediately after the digital presentation; thus, we do not know how the number of multimedia elements affects recall in the long term. Additionally, the digital presentation was relatively short (15 min) compared to an actual university lecture, raising questions about the extent to which the results can be generalized to a longer and hence more cognitively demanding lecture or seminar. Our measure of learning outcomes was a retention test and did not include transfer questions. Therefore, we can only generalize our results to the acquisition of fragmented knowledge rather than deeper understanding. To better understand the connection between multimedia information processing and attentional performance, it may be worthwhile to incorporate eye-tracking in the future. Mapping eye movements would help us to better understand the attentional mechanisms that contribute to successful learning with multimedia elements. Our sample was predominantly female, reflecting the typical gender distribution in psychology programs from which the participants were recruited. While this gender imbalance may limit the generalizability of our findings to populations with different gender compositions, based on previous research we believe that gender is unlikely to have influenced the main outcomes of this study (Bali et al., 2023a, 2023b). Another limitation concerns the lack of an explicit assessment of content difficulty. Although the content was evaluated by both students and lecturers, difficulty of the learning material may have varied across slides or topics. Future studies should include objective or subjective measures of content difficulty to discover its potential influence on learning efficacy. Content difficulty should be included as an additional independent factor. Despite these limitations, the advantage of the study is that the data were collected during actual seminar classes presenting theoretical material related to the curriculum. This increases both the ecological validity and the generalizability of our findings. Furthermore, we used a within-subject design, which allowed us to test improvements in the actual performance of the participants by comparing the achieved scores in different conditions. Also, instead of the media comparison approach that currently dominates the field (Buchner & Kerres, 2023), we followed the value-added approach and tested different versions of the same digital presentation with nuanced modifications. This allows us to make more precise suggestions about the optimal instructional design of this multimedia delivery mode (Baker et al., 2018).

### Conclusion

Visual multimedia elements in digital presentations can effectively enhance learning by reinforcing key content. However, the benefits of these elements depend on the learners’ attentional capacity. Research shows that university students can typically process up to three visual elements simultaneously, and sustained attention is crucial for efficient learning. These findings highlight the importance of considering both attentional and linguistic factors in multimedia instructional design. Future research should continue to investigate how attentional mechanisms interact with multimedia complexity and consider these factors not only in the context of seductive details to achieve a balance between engagement, clarity, and cognitive efficiency in learning environments. We believe that these results can guide educators in creating an instructional design that is consistent with the principles of multimedia learning while addressing the individual needs of their students.

## Data Availability

The datasets generated and/or analyzed during the current study are available in the Open Science Framework repository, https://osf.io/a7vh8/?view_only=736f6bcaa72d408fb3ace7ccee1d4aee.
